# Integrating domain-specific convolutional features with Swin Transformers for transparent cervical cancer diagnostics

**DOI:** 10.3389/fonc.2026.1878677

**Published:** 2026-08-26

**Authors:** Md. Abdur Rahman, Md Tofael Ahmed Bhuiyan, Md Fokrul Islam Khan, Md. Zia Uddin, Ulysse Teller Masao Côté-Allard, Abdul Kadar Muhammad Masum

**Affiliations:** 1 Computational Intelligence Lab, Southeast University, Dhaka, Bangladesh; 2 Computational Intelligence Lab, Dhaka, Bangladesh; 3 College of Business, Westcliff University, Los Angeles, CA, United States; 4 Sustainable Communication Technologies, SINTEF Digital, Oslo, Norway; 5 Department of Technology Systems, University of Oslo, Oslo, Norway; 6 Department of Computer Science and Engineering, Southeast University, Dhaka, Bangladesh

**Keywords:** cervical cancer screening, cross-modal attention fusion, cytopathology, deep learning, explainable AI (XAI), Swin Transformer

## Abstract

**Introduction:**

Cervical cancer screening relies heavily on manual cytopathological evaluation, which is subjective and prone to inter-observer variability. While deep learning offers automated alternatives, existing models often struggle to capture both global cellular context and fine-grained biological nuances. To address this, we propose CAMF-Swin, a novel hybrid architecture for precise cervical cell classification.

**Methods:**

The CAMF-Swin framework integrates a hierarchical Swin Transformer backbone with four domain-specific convolutional modules to explicitly extract morphological, textural, nuclear, and cytoplasmic features. A Cross-Modal Attention Fusion (CMAF) mechanism is utilized to dynamically synthesize these diverse representations. The model was trained using stratified five-fold cross-validation and an ensemble strategy. Performance was validated on the multi-class SipakMed dataset and evaluated for generalizability on an independent, highly imbalanced Mendeley Liquid-Based Cytology (LBC) dataset. Additionally, Local Interpretable Model-agnostic Explanations (LIME) were integrated for *post-hoc* visual interpretability.

**Results:**

The CAMF-Swin ensemble achieved a classification accuracy of 97.78% and a Matthews Correlation Coefficient (MCC) of 0.9722 on the SipakMed dataset. On the highly imbalanced Mendeley LBC dataset, the model maintained robust generalization, achieving 98.96% accuracy and an F1-Score of 0.9894. Furthermore, LIME visualizations confirmed the model’s reliance on clinically relevant biomarkers, successfully identifying features such as perinuclear halos in koilocytotic cells.

**Discussion:**

These results demonstrate significant performance superiority over standard Vision Transformer variants and traditional convolutional networks, highlighting the architecture’s resilience to real-world data imbalance. By providing transparent visual evidence of its decision boundaries, the model successfully bridges the gap between neural network predictions and expert pathological reasoning. Ultimately, the proposed framework establishes a robust foundational step toward automated clinical support, though rigorous prospective, multi-center validations remain necessary to facilitate safe real-world deployment.

## 1 Introduction

Cervical cancer is a common female cancer that is ranked fourth in the world (1). The cancer affects the part of the uterus that fuses with the vagina (2). Chronic infections with human papillomavirus are the most common oncogenic drivers. The presence of viruses interferes with cell metabolism and changes the morphology of cervical tissues (3). The number of cases is increasing in both developing and industrialized countries (4). The 2020 statistics indicate 604127 new cases and 341831 total deaths. This is the most diagnosed cancer in 23 different countries. It is the cause of death in 36 countries. The areas are sub-Saharan Africa, Melanesia, South America, and Southeast Asia (5).

Papanicolaou and HPV tests are among the main cervical screening tests. Cytology identifies precancerous lesions, and viral tests detect active infections (6). Medical imaging is an important and advanced concept in modern radiation therapy. The modalities commonly used for cancer treatment include computed tomography and magnetic resonance imaging (7). These imaging procedures give the necessary information on certain clinical outcomes. Magnetic resonance imaging is suitable for detecting parametrial invasion in cervical tissues (8). Improved diagnostic accuracy is achieved using three Tesla scanners and diffusion-weighted imaging. Clinicians like this modality for determining the local tumor extent (9). Moderate tissue is preferable to other soft-tissue resolutions because it facilitates accurate tumor size estimation. Imaging also helps identify a swollen lymph node in the pelvis (10).

To prevent errors in tumor interpretation, radiologists have to learn about staging (11). Moreover, the level of AI development will contribute significantly to the improvement of screening and cancer detection. These applications optimize staging systems and enhance clinical patient predictions. The use of integrated technology eventually helps the world in eliminating cervical cancer. It will take a lot of work to make AI a reliable tool among clinicians (12). Deep learning architectures, particularly Convolutional Neural Networks, have demonstrated robust performance across diverse medical image modalities, specifically excelling in cytopathological image segmentation and classification (13, 14). There are several cervical cancer algorithms that determine cervical cancer with the help of digital Pap smear images (15).

This study is based on an independent framework called CAMF-Swin. The offered architecture incorporates domain-specific extractors with Swin Transformer. Four expert convolutional modules obtain morphological and textual cytopathological features. Specialized segmentation units separate the nuclear and cytoplasmic compartments for analysis. The layers of cross-modal attention successfully combine five different feature vectors. Hierarchical windowing mechanisms extract multiscale information in cytology. The use of strategic data augmentation techniques can increase robustness based on SipakMed dataset. The ensemble technique is a strategy used in averaging predictions to minimize variance and error. The accuracy of this system is 97.78%. Visual explanations of AI applications, such as LIME, can provide clinicians with insights. The experimental results with CAMF Swin demonstrate that it outperforms the state of the art. This combined system has the benefit of overcoming major shortfalls in current cytology. High-quality statistical analysis demonstrates superior performance across several independent trials.

To summarize, the main contributions of this study are as follows:

- CAMF-Swin eliminates manual feature extraction through an automated pipeline.
- Domain-specific modules detect morphological nuances and diverse cellular textures.
- Cross-modal attention mechanisms integrate global semantic and local features.
- The ensemble approach ensures reliable performance across multiple independent folds.
- Proposed models exceed existing benchmarks in five class cytopathological tasks.
- Interpretability tools like LIME provide transparent insights into model decisions.
- Transfer learning achieves superior results by leveraging large-scale pretraining.

This research paper consists of eight distinct sections for comprehensive analysis. The second section evaluates past research and existing deep learning models. The third section describes the framework architecture and initial data preparation. This methodology outlines strategic data augmentation and advanced fusion strategies. The fourth section defines experimental methodology and various evaluation metrics. Detailed results and comparative analysis are presented in the fifth section. Section six explores model interpretability using tools like the LIME framework. The seventh section identifies minor limitations and also future research directions. Finally, the eighth section provides concluding remarks and summarizes the findings. This organized structure ensures a logical flow of the entire investigation.

## 2 Related works

Researchers developed ConvXGB to predict recurrence risks in cervical cancer (16). The study utilized multiparametric imaging from over 400 patients. ConvXGB surpassed clinical models for predicting survival within the cohort. This framework demonstrated a high area under the receiver operating characteristic curve. Limited dataset sizes and manual segmentation reduced overall model efficiency. Another study aimed to predict lymph node metastasis using radiomics (17). Investigators analyzed MRI scans from 153 patients. Special attention was given to T2-weighted images and ADC maps. A support vector machine classifier was trained on features selected by LASSO. The model achieved an AUC of 81.1% on the validation set. Clinical variables further improved the performance of this radiomics nomogram. Retrospective designs and small sample sizes hindered the broader generalization of results.

Laura et al. evaluated the prognostic significance of MRI-based ADC values (18). Normalizing tumor values to the myometrium improved disease-specific predictions. The traditional mean ratio achieved an overall accuracy of 68%. Differences in MRI protocols and vendors limited this work. Xue et al. proposed delta-radiomics to predict the severity of radiation proctitis (19). Radiomic features were extracted from images before and after radiotherapy. Logistic regression and LASSO techniques identified the most significant features. A random forest algorithm was utilized to develop the final model. The resulting model achieved an encouraging validation accuracy of 90%. Small single-center datasets and traditional machine learning methods restricted generalizability.

In study (20), the authors aimed to identify cervical cancer using MRI image datasets. There were 900 cancerous and 200 healthy images in the dataset. Four models, such as VGG16, CNN, KNN, and RNN, were compared. Well-developed preprocessing methods, such as standardization and noise removal, enhanced data quality. The VGG16 architecture was the highest in classification accuracy of 95.44%. Accurate predictions were achieved in other models, ranging from 86.23% to 92.3%. There were considerable imbalances in the datasets and variation in acquisition that limited the model’s performance on general measures. The reliance on the ready-made models also influenced the final diagnostic outcome. In the study of Qin et al., MRI was used on 392 clinical patients (21). Scholars used deep multiple-instance learning to forecast lymph node metastasis. The ResNet-50 architecture was used to extract features without manual tumor annotation. This was an automated method that attained an external validation score of 76.5. The combination of clinical parameters developed a hybrid model that performed better. This integrated model achieved a training-cohort rating of 83.8. A retrospective study and a limited sample size were also major study limitations. These aspects could have introduced selection bias into the reported outcomes.

MRI is the known gold standard of local cervical staging (22). This modality provides better contrast for assessing stromal infiltration and size. T2-weighted high-resolution imaging is useful in detecting local parametrial invasion. The negative predictive values were reported to be about 94-95% accurate. Functional imaging enhances the sensitivity of detecting malignant pelvic lymph nodes. Among the notable weaknesses are increased prices and much longer scanning times. Magnetic resonance-positron emission scans are the best combination of the two to optimize clinical treatment planning. The chemoradiotherapy response using pre-treatment MRI datasets was also predicted by researchers (23). The study examined 252 patients with respect to imaging characteristics. Deep learning radiomics performed better at prediction than conventional handcrafted models. The 3D convolutional network had more than 73% classification accuracy. The overall predictive ability of models was also enhanced by the addition of clinical variables. Smaller cohort sizes and the lack of external validation affected generalizability.

A single study aimed to identify cervical cancer through multiparametric MRI (24). This paper has designed a radiomics model to predict lymph vascular space invasion. Significant feature selection was performed using T2-weighted and diffusion-weighted imaging. The identification of 13 important imaging features was performed using regression analysis within the patient cohort. The resulting nomogram achieved an 83% AUC in the test groups. The first training yielded a classification accuracy of 78%. Other studies have explored advanced computer-aided diagnosis (CAD) methods to enhance cervical cancer detection across both CT and MRI modalities (25). Recent approaches employ ensemble learning to reduce misclassification between benign and malignant tumors. For example, investigators developed a novel methodology combining a Large Vision Model (LVM) for fine-grained spatial features with InternImage (based on InceptionV3) to extract pre-trained deep, tumor-specific patterns. To avoid overreliance on a single model, the Shark Optimization Algorithm (SOA) was implemented to dynamically select optimal weight parameters. This ensemble model effectively classified images into normal, benign, and malignant categories, achieving an outstanding diagnostic accuracy of 98.49% for CT images and 92.92% for MRI images. **Table 1** summarizes recent studies on cervical cancer, including the methods used, their limitations, and the performance metrics reported. To shift the focus toward direct cytopathological analysis, several recent studies have exclusively emphasized deep learning methodologies for Pap smear and liquid-based cytology classification. For instance, Albzour and Lam (37) systematically optimized a lightweight Vision Transformer (ViT-Tiny) architecture on the Herlev Pap smear dataset, achieving a cross-validation accuracy of 95.15% while utilizing Grad-CAM to provide clinical interpretability that strongly aligns with morphological cytopathology. In another recent study, researchers applied transfer learning via the InceptionV3 architecture specifically to the SipakMed dataset, attaining a robust 94.89% multi-class accuracy for automated cervical cell classification (38) (1). Addressing the integration of feature extraction and classification, Mathivanan et al. (39) proposed a CNN-VGG16 framework evaluated on the SIPaKMeD benchmark, achieving an impressive 99.35% for five-class classification by optimizing convolutional feature mappings (2). Furthermore, Cömert et al. (40) advanced cytology classification by integrating Convolutional Block Attention Modules (CBAM) into parallel convolutional branches, significantly enhancing the model’s ability to focus on informative spatial areas of cervical cells (3). These cytology-centric innovations highlight the urgent clinical necessity for architectures that natively balance global structural comprehension with fine-grained cellular nuance, a gap that is directly addressed by our proposed CAMF-Swin framework.

**Table 1:** Analysis of earlier research on cervical cancer.

Ref	Methodology	Limitations	Performance
(16)	ConvXGB (CNN + XGBoost) for recurrence risk prediction	Small dataset, data variability	AUC 0.872 (1-year RFS), AUC 0.882 (3-year RFS)
(17)	Radiomics-based SVM with LASSO for lymph node metastasis (LNM) prediction	Retrospective study, MRI protocol variability, small sample size	AUC 0.804 (train), AUC 0.811 (validation), C-index 0.916
(18)	ADC normalization to myometrium ADC for disease-specific survival (DSS) prognosis	Heterogeneous MRI protocols, retrospective design	Accuracy 0.68 (DSS prediction)
(19)	LASSO and Random Forest	Small sample size	Accuracy 0.90 (validation)
(20)	VGG16, CNN, KNN, RNN with preprocessing (standardization, normalization, noise reduction)	Dataset imbalance, reliance on pretrained models, MRI acquisition variations	VGG16: 95.44%, CNN: 92.3%, KNN: 89.99%, RNN: 86.23%
(21)	D-MIL model (ResNet50) for LNM prediction	Small dataset, retrospective bias	AUC 83.8% (train), 76.4% (internal), 83.5% (external)
(22)	MRI as a gold standard for cervical cancer staging	High cost, long scan time	Accuracy 88%, Sensitivity 86%, Specificity 84%
(23)	Handcrafted radiomics (SVM) vs. deep learning radiomics (3D CNN) for CRT response prediction	Small sample size, no external validation	HCR Accuracy 59.8%, DLR Combined Accuracy 77.7%
(24)	Radiomics-based nomogram using mRMR and LASSO	Single-institution data, MRI variability, manual segmentation	Accuracy 78% (train), 72.2% (test), AUC 0.838 (train), 0.837 (test)
(25)	Ensemble of Large Vision Model (LVM) & InternImage optimized with Shark Optimization Algorithm (SOA)	Validated primarily on specific regional, single-institution datasets (KAUH)	CT Accuracy: 98.49%, MRI Accuracy: 92.92%

Earlier research tends to rely on manual classification and small datasets. The classical machine learning models are often limited in their generalizability across cohorts. Diagnostic accuracy is restricted by using off-the-shelf architecture without domain adaptation. Retrospective designs and protocol variations bring about selection bias and inconsistencies. Numerous current frameworks lack transparency into clinical decision support systems. Large class imbalance is a disadvantageous factor that hinders the performance of standard convolutional networks. The limitations are defeated by the automation of the proposed CAMF Swin architecture.

Based on the critical analysis of prior studies, several major research gaps in automated cervical cytopathology currently remain:

- Domain-Agnostic Architectures: Existing models frequently utilize off-the-shelf generic neural networks lacking domain-specific adaptation. Consequently, they struggle to simultaneously capture global cellular semantics and fine-grained biological nuances.
- Restricted Generalizability and Class Imbalance: Many studies depend on small, single-center datasets or fail to adequately address severe class imbalances. This reliance leads to selection bias and restricts model generalizability across diverse clinical cohorts and scanning protocols.
- Manual Processing Bottlenecks: A recurring dependence on manual feature extraction, manual lesion segmentation, or handcrafted radiomics limits the scalability and automation of current diagnostic pipelines.
- Lack of Clinical Transparency: High-performing deep learning frameworks often operate as “black boxes.” Without the integration of Explainable AI (XAI) to map decisions back to pathological evidence, these systems fail to garner the trust required for clinical adoption.

Domain-specific extractors are those that extract biological subtleties that generic models overlook. Multi-scale feature extraction models are enhanced by hierarchical window attention mechanisms. Global semantic information is optimized via integrated cross-modal fusion layers. Automation eliminates human interpretation and minimizes interpretive errors. Diagnostic trust requires clinical transparency that is guaranteed by tools such as LIME. The best performance is verified by the highest accuracy of 97.78%. Strong ensemble methods provide a robust solution to overfitting and enhance model stability.

## 3 Methodology

**Figure 1:**
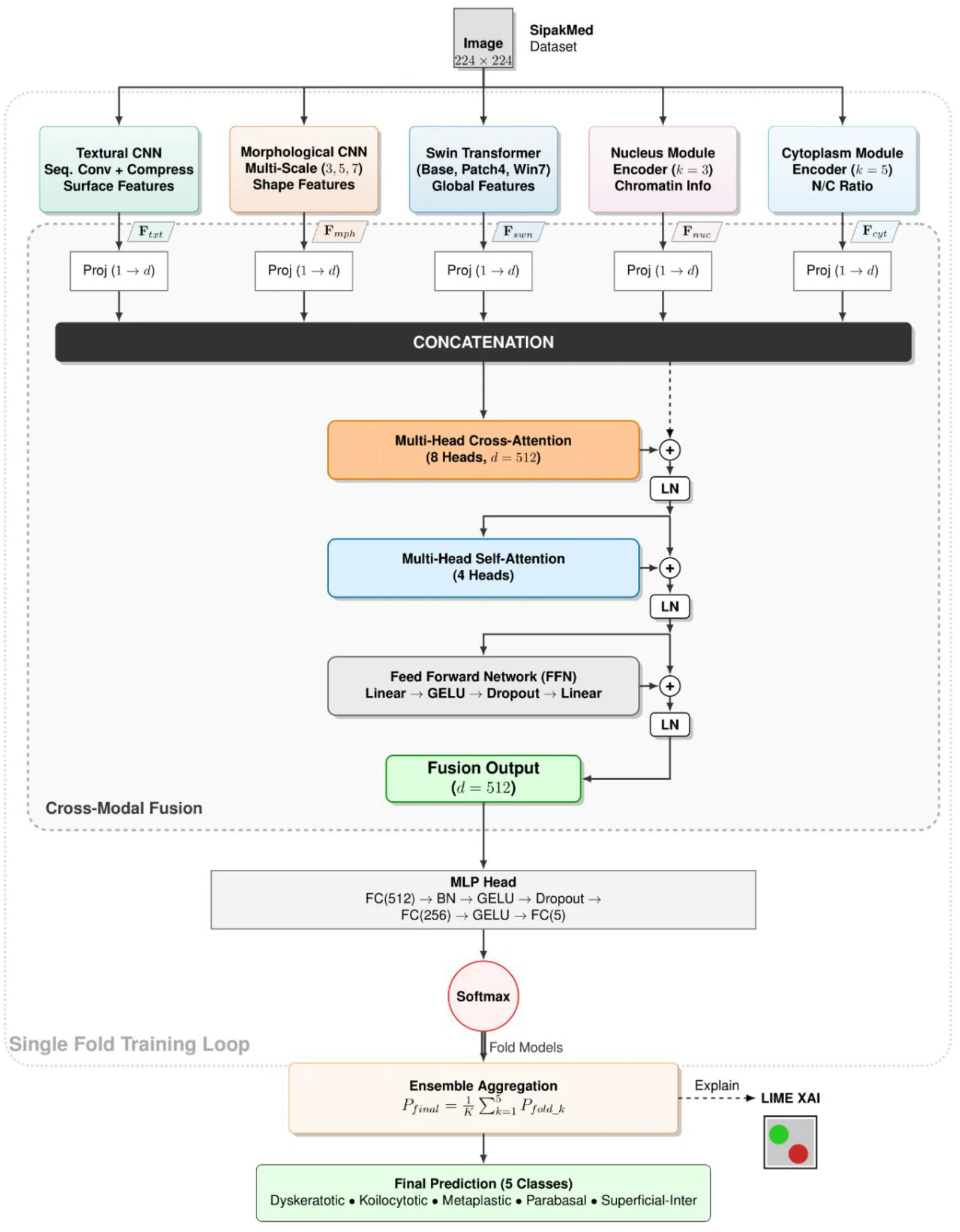
Schematic overview of the proposed CAMF-Swin architecture for cervical cell classification.

The proposed CAMF-Swin architecture incorporates Swin Transformer and domain-specific extractors. The images of SipakMed cervical cytology are resized to the same resolution. Massive data augmentation methods strengthen training and reduce overfitting. The Swin backbone makes use of the pretrained weights of ImageNet-22K database. Parallel convolutional modules are used to extract morphological, textural, nuclear, and cytoplasmic features. These five different feature vectors are crossed in a cross-modal attention layer. These generated embeddings communicate with each other via multi-head cross-attention and self-attention. The final fused vector goes through a multilayer perceptron classification head. The model uses cross-validated stratification, including five-fold cross-validation, during training. The AdamW optimizer is a loss minimizer that uses cross-entropy loss with dynamic scheduling. Ensemble strategies take averages of predictions to decrease variance and enhance performance. The measures of evaluation are accuracy and the score of the Matthews Correlation Coefficient. The Local Interpretable Model-agnostic Explanations framework has been successfully used for *post-hoc* interpretability. Visualization of the salient region provides clinical transparency to the final predictions. This methodology pipeline has guaranteed high performance and clinical transparency. **Figure 1** represents the general structure of the given CAMF-Swin architecture.

### 3.1 Dataset and preprocessing

To ensure dimensional consistency across the diverse multi modal feature extraction modules, all original cytological images were uniformly resized to a fixed target resolution of 224×224 pixels prior to training. Following this standardization, a rigorous and heavily stratified data partitioning strategy was employed to facilitate robust model evaluation and prevent data leakage. The complete dataset was first divided by reserving 20% of the images as a strictly independent testing set, intended exclusively to evaluate the final ensemble generalization. The remaining 80% of the dataset was utilized for model development via a stratified 5-fold cross validation framework and the securely held out 20% was exclusively retained for the ultimate unbiased performance assessment of the completed ensemble model.

#### 3.1.1 The SipakMed dataset

This study explores the publicly available SipakMed dataset that is used to evaluate it (26). The data are digitized images of individual cervical cell samples. The basic Image data comprises five cytopathological diagnostic categories. These are dyskeratotic, koilocytotic, metaplastic, parabasal, and superficial cells. Every image is resized to a standardized resolution. Preprocessing changes the original BGR color space to the RGB color space. The OpenCV library is useful in loading and iterating through the directories efficiently. The use of standardized input dimensions remains critical for uniform deep learning processing. The multi-class design reflects clinical decision-making in cytopathology. Thus, this criterion can be used successfully to assess the work of classification models.

#### 3.1.2 Independent Mendeley LBC dataset

A publicly available benchmark dataset provides the basis for developing predictive algorithms. Data derived from hospital-based, real-world clinical environments is also highly important because it enables automated medical research and disease diagnosis and ensures that the resulting algorithms achieve high accuracy in a regional context. The proposed model was tested on a different independent dataset, a strategy used to measure the model’s generalization ability. For this purpose, the Mendeley dataset (27) was used. It is important to explicitly state that our evaluation on the Mendeley LBC dataset constitutes an independent training and testing pipeline, rather than a zero-shot cross-dataset transfer of the weights learned from the SipakMed dataset. To accommodate this dataset’s specific taxonomy, the terminal classification head of the CAMF-Swin architecture was adjusted to output four classes prior to training. Liquid-based cytology is an existing, standard cervical screening test, and the repository lends itself to research in image segmentation and classification. The dataset includes 963 images from 460 patients, acquired at 40X magnification using a Leica ICC50 HD microscope, with samples prepared by liquid-based cytology techniques. It is divided into four classifications, namely, precancerous and cancerous lesions in accordance with The Bethesda System, although the distribution is quite unbalanced: 613 images are negative for intraepithelial malignancy, 163 represent high-grade squamous intraepithelial lesion, 113 represent low-grade squamous intraepithelial lesion, and 74 represent squamous cell carcinoma. Microscopic examination of abnormal cellular alterations allows detection of malignancy; however, it is manual, time-consuming, and prone to inter-observer variability. Computer-assisted diagnosis holds the promise of improving diagnostic throughput, thereby allowing earlier therapeutic intervention and reducing the risk of late diagnosis. All preprocessing procedures applied to this dataset are the same as those used for the SipakMed dataset.

#### 3.1.3 Data augmentation and transforms

Strict augmentation pipeline guarantees that the model is better generalized (28). Training variations bring a lot of variation in the input image set. Up to a random angle of 25, the rotation is orientation-invariant. Random resized cropping is used to train at different microscope magnifications. Horizontal and vertical flips enhance resistance to symmetric cellular structures. Color jittering introduces perturbations in brightness, contrast, and saturation. These particular changes resemble variations of inherent staining in clinical cytology. Augmentation operations take a fixed probability parameter of 0.5. Training is performed using standard ImageNet statistics for pixel values. In validation sets, deterministic resizing and normalization are used to ensure consistent evaluation. This will be a good measure to counter overfitting in the high-capacity network. The network acquires inherent cytopathological properties that are independent of preparation effects. Preprocessing is performed in a standard manner to ensure models are evaluated fairly and consistently across folds. These powerful transformations are useful for stabilizing and accelerating the training of neural networks.

### 3.2 Architecture: the CAMF-Swin model

**Figure 2:**
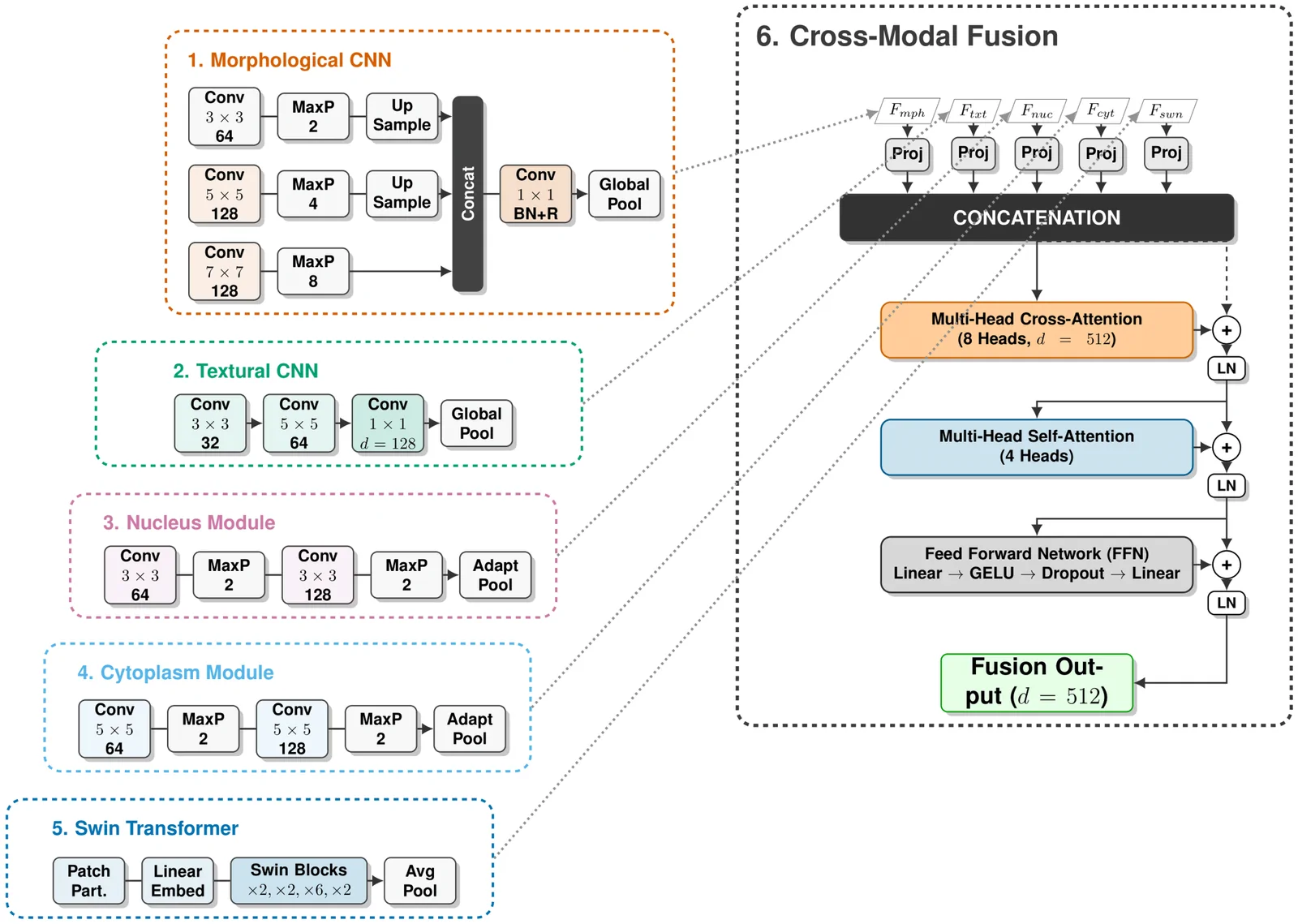
Detailed architectural design of the five feature extraction streams and the internal mechanism of the Cross-Modal Fusion module.

The CAMF-Swin framework combines the Swin backbone with dedicated extractors (29). The hybrid model incorporates already trained weights and hierarchical window attention. The last transformer blocks are then fine-tuned to retain valuable generic visual cues. Four parallel convolutional modules are used to simultaneously produce morphological and textural features. Multi-scale kernels represent cell shapes, whereas sequential layers learn textures. Specialized segmentation units isolate cytoplasmic and nuclear centers of analysis. Each of the four auxiliary extractors is effective in creating compact single-output feature vectors. Five separate feature streams are combined in a cross-modal attention layer. The interaction between projected embeddings is based on attention and dynamic self-modulation. This fusion process enables the fusion of other representations by each feature type. The final fused vector is fed into a multilayer perceptron classification head. Gradient flow is also supported by residual connections and GELU activations. The last output layer is feature-to-five diagnostic cytological classes. PyTorch framework is used in the implementation to provide effective deep learning processing. This methodology pipeline supports high performance and clinical transparency in general. The architectural design of the five streams of feature extraction and the interior design of the Cross-Modal Fusion module is given in **Figure 2**.

#### 3.2.1 Backbone: Swin Transformer

The model utilizes a Swin Transformer base as the primary backbone. This architecture was pretrained on the very large ImageNet-22K dataset (30). It introduces a hierarchical, shifted-window mechanism for local self-attention. Successive windows shift to create essential spatial connections during processing. This design captures both fine local details and broad global contexts. The configuration employs a patch size of four for input images. To fully leverage the robust feature representations acquired during pre-training on the expansive ImageNet-22K database, a selective partial fine-tuning strategy is employed. Deep hierarchical networks intrinsically capture universal, low-level visual primitives such as structural edges, color gradients, and fundamental textures in their early and intermediate layers. To strictly preserve these generic visual features and prevent catastrophic forgetting on the smaller target dataset, the gradients of all early Swin Transformer blocks are frozen. Conversely, only the parameters of the final two transformer blocks are unfrozen and updated via backpropagation. This paradigm allows the frozen layers to act as a highly stable, generic feature extractor, while the unfrozen terminal blocks dynamically map these foundational visual primitives to highly complex, domain-specific cytological morphologies required for cervical cancer classification. The backbone generates a semantic feature vector for each cell image. This vector encapsulates complex information regarding the entire cervical cell structure. Hierarchical structures produce various feature maps across different network depths. Efficiency and structural depth provide key advantages for the classification task. The extracted features serve as the primary stream for subsequent fusion.

### 3.3 Domain-specific feature extractors

Four dedicated convolutional modules efficiently extract specific streams of cytopathological features. These streams can capture biological subtleties that the transformer backbone may miss. The morphological extractor utilizes a parallel line to extract shape information. Three, five, and seven kernel sizes provide a wide spatial detail. Bilinear interpolation makes all the feature maps to have the same spatial dimensions. 1×1 convolutions are used to reduce the concatenated feature maps to smaller vectors. The textural extractor is oriented toward using sequential convolutional layers to focus on chromatin patterns. Global average pooling yields a fine, concise feature vector. The nucleus segmentation module separates the nuclear region through small kernels. These 3 × 3 kernels provide the high-frequency edges of the nuclear boundary. The cytoplasm module performs an analysis of the surrounding area with 5 × 5 kernels. The larger kernels reflect a wider range of low-frequency intensity variation in the cytoplasm. Each of the four auxiliary extractors gives compact 1-dimensional output vectors to be fused. Each module is trained to learn distinct biological representations in an end-to-end manner. This domain design guarantees a clear and solid classification of cells.

#### 3.3.1 Morphological feature extractor

In Equation 1, the variable X represents the standardized multi-channel color pixel matrix of the input cervical cytology image. The function Conv denotes the spatial convolutional filter traversing the image to detect structural boundaries. The variables H_1_, H_2_, and H_3_ represent the multi-scale morphological feature maps generated at different receptive fields, effectively capturing cellular shape variations ranging from local edge details to the broader cellular outline. The symbol sigma denotes the Rectified Linear Unit activation function introducing non-linearity, and Pool signifies the adaptive average pooling operation that standardizes the spatial dimensions of these biological shape maps.

(1) $$\begin{array}{c} H_{1}\;=\;Pool\left(\sigma \left(Conv_{3\times 3}\left(X\right)\right)\right)H_{2}\\ =\;Pool\left(\sigma \left(Conv_{5\times 5}\left(X\right)\right)\right)H_{3}\\ =\;Pool\left(\sigma \left(Conv_{7\times 7}\left(X\right)\right)\right) \end{array}$$

where σ is the ReLU activation function. Adaptive average pooling is known as the Pool operation. In this pooling step, the spatial size of each output is standardized. The H_1_ and H_2_ have reduced spatial dimensions compared to H_3_. Bilinear interpolation upsamples H_2_ and H_1_ to H_3_. This alignment is such that all feature maps have the same height and width. The three aligned feature maps are then concatenated along the channel dimension. 1×1 convolution is applied to the concatenated multi-scale feature volume. This convolution not only down-sampling the channel but also combines information on different scales. This compressed volume is finally summed in a global average pooling layer. Combines spatial data into one feature. The resultant value is a small 256-dimensional vector. This cell morphological signature is captured in the vector Fmorph. The extractor enables the specific modeling of shape traits that are insensitive to scale. These properties are essential to proper classification using cytopathology.

#### 3.3.2 Textural feature extractor

In Equation 2, the input variable X is the raw cytopathological image matrix. The consecutive Conv functions extract increasingly complex structural patterns. The global average pooling operation collapses all spatial information into a single vector. The resulting parameter Ftext is a dense mathematical representation explicitly encoding the chromatin distribution and local granularity of the cell.

(2) $$F_{text}\;=\;GAP\left(Conv_{1\times 1}\left(Conv_{5\times 5}\left(Conv_{3\times 3}\left(X\right)\right)\right)\right)$$

In this case, GAP refers to the global average pooling. The last 1×1 convolution is channel-wise refining features. Such a step permits adaptive re-weighting of the varying textural channels. It then collapses all spatial information from a global average pooling operation into a vector. This operation guarantees the invariance to the spatial position of textural patterns. The result is a small F_text_ 128-dimensional feature vector. This is a Vector that expressly describes the cell chromatin distribution and texture. These textual features are very important diagnostic clues in cytopathology. The extractor offers a specific avenue of this important biological antecedent.

#### 3.3.3 Nucleus and cytoplasm segmentation modules

In Equations 3 and 4, the input variable X denotes the cellular image matrix, while σ represents the non-linear activation function and BN denotes batch normalization for stabilizing pixel intensity distributions. The Nuclear Feature Extraction Module employs compact 3×3 convolutional kernels to preserve a localized receptive field that is highly sensitive to high-frequency spatial transitions. This design enables precise detection of sharp nuclear membrane boundaries and granular chromatin patterns, allowing the resulting feature vector F_nuc_ to encode critical information related to nuclear edge contrast and internal chromatin density. In contrast, the Cytoplasm Segmentation Module utilizes 5×5 convolutional kernels with a spatial padding of two to preserve feature map dimensions while expanding the receptive field. By analyzing a 25-pixel neighborhood, the module captures broader spatial context, enabling effective aggregation of gradual intensity variations, diffuse textural patterns, and staining gradients that characterize the cytoplasm. Consequently, the output feature vector F_cyto_ encodes the expansive low-frequency color gradients and diffuse morphological characteristics of the surrounding cellular region, providing complementary information essential for reliable cell classification.

(3) $$F_{nuc}\;=\;Pool\left(\sigma \left(BN\left(Conv_{3\times 3}\left(X\right)\right)\right)\right)$$

In this case, σ represents the ReLU activation, and BN represents batch normalization. Adaptive max pooling is referred to as the Pool operation. This obtains a 128-dimensional nuclear feature vector, Fnuc. Instead, the Cytoplasm Segmentation Module uses the 5×5 convolutional kernels. These bigger kernels are able to record wider ranges of variations of intensity in the cytoplasm. The main targets are lower-frequency texture and staining patterns. Its transformation follows an identical functional structure, which is formally delineated in Equation 4:

(4) $$F_{cyto}\;=\;Pool\left(\sigma \left(BN\left(Conv_{5\times 5}\left(X\right)\right)\right)\right)$$

Its result is a clear 128-dimensional F_cyto_ vector. Both modules operate independently of each other. They are set with various random weights and trained individually. This promotes specialization to their subcellular structures. The nuclear vector is directed at the membrane and chromatin properties. The cytoplasmic texture and intensity of cytoplasmic color are denoted by the cytoplasmic vector. Such clear biological priors are much more interpretable of the model.

### 3.4 Cross-modal attention fusion

The Cross-Modal Attention Fusion (CMAF) module integrates five heterogeneous feature vectors derived from the Swin backbone and the four domain-specific extractors. To guarantee alignment within a shared semantic space, each vector is mapped to a uniform 512-dimensional latent representation via dedicated linear projection layers.

The projection is defined for each feature vector F_i_ as demonstrated in Equation 5:

(5) $$E_{i}=W_{i}F_{i}+b_{i}$$

In Equation 5, the term Fi represents a specific biological feature stream extracted from the preceding modules, such as the morphological shape vector or the nuclear boundary vector. The parameter Wi is the projection weight matrix, and bi is the learnable bias term. These parameters act as a linear transformation mechanism mapping the diverse biological traits into a uniform semantic space, generating the aligned embedding E_i_. The dimension parameter dmodel dictates the uniform size of this shared latent space. In Equation 6, the matrix Q acts as the query, representing the target biological cell component currently seeking contextual diagnostic information. The matrix K represents the key, containing the encoded signature of all other available cellular modalities. The matrix V holds the value, which corresponds to the actual extracted cytological feature data. The term dk is the scaling factor based on the dimension size, which prevents the gradient from saturating when computing the diagnostic correlation between different cellular components during the fusion process.

The scaled dot-product attention mechanism is applied as follows in Equation 6:

(6) $$Attention\left(Q,K,V\right)=softmax\left(\frac{QK^{T}}{d_{k}}\right)V$$

Cross-attention enables each feature type to attend to all others (31). This allows dynamic modulation and contextual integration of information. A residual connection and layer normalization stabilizes the output features. A second multi-head self-attention layer further refines this representation. This layer performs standard self-attention on the modulated sequence. Another residual connection and layer normalization follow this step.

The refined sequence is then processed by an internal feed-forward network (FFN). To maintain smooth gradient flow and handle the complex fused representations, this network utilizes a Gaussian Error Linear Unit (GELU) activation function, which is applied strictly between the FFN’s expansion and compression linear layers, followed by a dropout layer. Finally, average pooling is applied across the sequence dimension. This collapses the five-modality sequence into a single holistic vector. The output is the final fused representation F_final_ for classification.

#### 3.4.1 Projection

The projection step aligns five distinct feature vectors into a common space. Each feature vector originates from a different component of the model. The Swin backbone provides a high-level semantic feature vector F_swin_. The morphological extractor outputs a shape-focused vector F_morph_. The textural extractor produces a chromatin-pattern vector F_text_. The nucleus module yields a nuclear-specific vector F_nuc_. The cytoplasm module generates a cytoplasmic feature vector F_cyto_.

Each vector inherently possesses a different original dimensionality and statistical distribution due to the heterogeneous nature of the extraction modules and the distinct biological properties they encode. Architecturally, the generic Swin backbone yields a high-dimensional semantic vector, while the domain-specific convolutional extractors are mathematically compressed to lower dimensions, specifically 256 for morphological features and 128 for textural, nuclear, and cytoplasmic features, to optimize feature density. Statistically, these vectors diverge significantly because they capture fundamentally different cytological characteristics; for example, the nuclear module encodes high-frequency, sparse spatial transitions, whereas the cytoplasm module processes dense, low-frequency color gradients. Furthermore, the reliance on LayerNorm within the transformer backbone versus batch normalization within the parallel convolutional pipelines introduces inherently divergent activation scales and shifts. To reconcile this structural and statistical heterogeneity, a dedicated linear transformation projects every vector into a unified, normalized semantic latent space of dimension *d_model_*. This common fusion dimension is set to 512.

To compute contextual interactions across diverse modalities, the scaled dot-product attention mechanism strictly requires all input tensors to share a homologous dimensionality. However, our extracted feature vectors inherently possess heterogeneous original dimensions (*d_i_*), such as 256 for morphological features and 128 for textural, nuclear, and cytoplasmic features. The dimension parameter *d_model_* (empirically set to 512) dictates the uniform size of the shared latent space by strictly defining the target output dimension for the modality-specific linear projections. This operation is mathematically defined for each feature stream *i* in Equation 7:

(7) $$E_{i}=W_{i}F_{i}+b_{i}$$

In this formalization, $W_{i}\in {\mathbb{R}}^{d_{model}\times d_{i}}$ serves as a learnable weight matrix explicitly tailored to the original dimensionality of the *i*-th feature stream ( $F_{i}\in {\mathbb{R}}^{d_{i}}$), and $b_{i}\in {\mathbb{R}}^{d_{model}}$ is the corresponding learnable additive bias vector. By projecting *F_i_* through *W_i_*, varying dimensionalities are mathematically transformed into a unified 512-dimensional semantic space. Consequently, this geometric alignment ensures that all resulting projected embeddings ( $E_{i}\in {\mathbb{R}}^{d_{model}}$) are dimensionally compatible, establishing the requisite mathematical foundation for calculating cross-modal attention.

#### 3.4.2 Multi-head cross-attention

The single sequence S is fed into a multi-head attention block. In this block, the first layer is a cross-attention layer applied to the five modalities. All query-key and value matrices result from the same sequence S. The attention mechanism computes interactions between all pairs of feature streams. This enables the various modalities to contextualize themselves relative to one another. The process has eight parallel attention heads to provide increased capacity. Each head transforms the input into the query key and value spaces, respectively. All head attention is concatenated and projected linearly. The residual connection combination leaves the original input sequence in this output. The resulting feature distribution is then stabilized by using layer normalization.

The process for the cross-attention step is formally expressed in Equation 8:

(8) $$Z_{1}\;=\;LayerNorm\left(S\;+\;MHA_{cross}\left(S,\;S,\;S\right)\right)$$

A subsequent multi-head self-attention layer refines this modulated sequence. This layer also employs the standard self-attention mechanism internally. It allows features to further integrate information within the fused context. Another residual connection and layer normalization follow this operation, as formulated in Equation 9:

(9) $$Z_{2}\;=\;LayerNorm\left(Z_{1}\;+\;MHA_{self}\left(Z_{1},\;Z_{1},\;Z_{1}\right)\right)$$

The final output is a coherent sequence of five refined feature embeddings. These embeddings now contain rich cross-modal contextual information.

### 3.5 Classification head

The final fused feature vector (*F_final_*) is passed to the multilayer perceptron classification head, where GELU activations are extensively utilized to map complex non-linear decision boundaries. The classification head process unfolds in four distinct stages. First, the fused features pass through an initial linear layer mapping to a 512-dimensional space, immediately followed by a GELU activation, batch normalization, and dropout. Second, a parallel residual dense layer processes the original fused features via a linear transformation without initial activation. Third, this residual vector is summed with the output of the first main layer, and a second GELU activation is applied to this combined representation to ensure non-linear integration of the skip connection. Finally, the tensor passes through a second dense layer reducing the dimensionality to 256, followed by a third GELU activation, batch normalization, and dropout, before the final linear layer projects the features to the five diagnostic classes.

This integrated residual design ensures robust gradient flow during the training process, functioning as a critical countermeasure against the vanishing gradient problem commonly encountered in deep fine-tuning. By providing a direct structural skip connection, the residual pathway allows the backpropagated error signal to bypass the complex, non-linear activation bottlenecks of the primary dense layers, delivering an unattenuated gradient directly to the Cross-Modal Attention Fusion module. When synergistically coupled with our selective partial fine-tuning strategy, which freezes early Swin Transformer blocks to drastically shorten the overall backpropagation path, this residual architecture guarantees that the unfrozen terminal layers receive a stable and mathematically potent gradient. Consequently, it mitigates potential optimization failures in deep fine-tuning before the output logits are converted to probabilities via softmax normalization, where the class with the highest probability is ultimately selected as the final prediction.

The entire classification process can be represented stepwise as detailed in Equation 10:

(10) $$\begin{array}{c} x\;=\;Dropout(BN(GELU(W_{1}F_{final}\;+\;b_{1}))))r\;\\ =\;W_{res}F_{final}\;+\;b_{res}x\;=\;GELU\left(x\;+\;r\right)x\\ =\;Dropout\left(BN\left(GELU\left(W_{2}x\;+\;b_{2}\right)\right)\right)output\\ =\;W_{o}x\;+\;b_{o} \end{array}$$

This residual design ensures robust gradient flow during training. It mitigates potential vanishing gradient problems in deep fine-tuning. The output logits are converted to probabilities via softmax normalization. The class with the highest probability is selected as the final prediction.

## 4 Experimental methodology

To guarantee absolute algorithmic reproducibility and facilitate independent verification of the proposed CAMF-Swin framework, strict deterministic controls and comprehensive software environment details were enforced throughout all experimental phases. A universal random seed of 42 was systematically applied across the core Python environment, the NumPy random number generator, and both the CPU and CUDA functions within PyTorch. Furthermore, the PyTorch cuDNN backend was explicitly configured to operate in deterministic mode with benchmarking disabled to eliminate non-deterministic algorithmic selections during hardware acceleration. The end-to-end computational pipeline was developed primarily using PyTorch, while the generic Swin Transformer backbone was instantiated through the ‘timm’ library. Ancillary data processing and performance evaluation were performed using OpenCV, Pandas, and Scikit-learn, whereas visual interpretability analysis was conducted using the LIME package. The automated preprocessing pipeline standardized all raw cytological images by converting them from the BGR color space to the RGB color space, uniformly resizing every image to a resolution of 224 × 224 pixels, and applying channel-wise normalization using the ImageNet statistical priors with mean values of 0.485, 0.456, and 0.406 together with standard deviation values of 0.229, 0.224, and 0.225. During training, the preprocessing pipeline incorporated dynamic data augmentation through random rotations of up to 25°, random resized cropping, horizontal and vertical flipping, and comprehensive color jittering to vary brightness, contrast, and saturation. The domain-specific morphological, textural, nuclear, and cytoplasmic feature extractors were implemented as custom PyTorch modules fully integrated into the end-to-end backpropagation framework rather than executed as separate offline processing components.

### 4.1 Training protocol

A stratified five-fold cross-validation strategy is used to train the model. This preserves the original dataset’s class distribution in each fold. The cross-entropy loss is used to optimize the network parameters. This loss is the difference between the forecasts and the actual labels. The mathematical formula of a one-sample calculation is presented in Equation 11:

(11) $${ℒ}_{CE}\;=\;-\sum_{c=1}^{5}y_{c}\;log\left(p_{c}\right)$$

In Equation 11, the parameter *y_c_* designates the clinically verified ground truth label for a specific diagnostic class from the SipakMed dataset. The parameter pc represents the softmax probability output calculated by the model, signifying the network confidence that the input cell image belongs to that particular cytopathological class. The initial learning rate is 1 × 10^-4^. The weight decay is set at 1 × 10^-5^. ReduceLROnPlateau is a learning-rate scheduler that dynamically adjusts the learning rate. Scheduler evaluates the validation loss measure at the end of every epoch. On the plateau, the learning rate decreases by a factor of 0.3. The set is patient for seven epochs before any reduction in the rate is realized.

Early termination of training occurs when there are 5 consecutive stagnant epochs on the validation set. This avoids overfitting, and it saves on computation. The mini-batch size used in training is sixteen images. The maximum number of epochs per fold on the model is twenty. The pipeline as a whole is implemented using PyTorch and associated libraries.

### 4.2 Evaluation metrics

To ensure absolute fairness and reproducibility in our comparative analysis, the numerical values for all evaluation metrics across the various baseline networks and the proposed CAMF architectures were computationally derived using an identical, highly standardized execution pipeline. During the inference phase on the strictly held-out test sets, the raw output logits from each respective model were first processed through a softmax activation function to yield a continuous probability distribution across the five cytopathological diagnostic classes. For the calculation of discrete performance measures, specifically Accuracy, macro-averaged and weighted-averaged Precision, Recall, F1-Score, the Matthews Correlation Coefficient, and Cohen’s Kappa, these continuous probabilities were converted into definitive class predictions via an argmax operation and subsequently evaluated against the clinically verified ground truth labels using deterministic Scikit-learn backend functions. Conversely, for threshold-independent evaluations such as the Area Under the Receiver Operating Characteristic curve, the continuous probability arrays were utilized directly within a One-versus-Rest multiclass framework to calculate the final cumulative score. Furthermore, the final performance values reported for the CAMF-Swin ensemble were obtained by mathematically averaging the multi-class softmax probability vectors generated by all five independently trained fold models prior to the final argmax classification step. This rigorous, unified computational approach guarantees that the variance in the reported metrics across all tables is strictly indicative of inherent architectural capabilities rather than discrepancies in evaluation methodologies.

Cohen’s Kappa statistic measures inter-rater agreement against chance. This metric is particularly valuable for imbalanced medical datasets. It is mathematically defined in Equation 12:

(12) $$\kappa \;=\;\frac{p_{o}\;-\;p_{e}}{1\;-\;p_{e}}$$

where p_o_ is observed agreement and p_e_ is expected agreement. It is shown to be the Area Under the Receiver Operating Characteristic. The Multiclass evaluation strategy employed at AUC-ROC is One-versus-Rest. The average AUC across the five classes yields a final cumulative score. Together, these measures provide a comprehensive and impartial performance appraisal.

### 4.3 Ensemble strategy

An ensemble technique is a combination of the results of all five-fold cross-validations (32). The approach reduces variance and improves generalization on test sets. Complementary data subsets are trained using five different models. Each model provides a probability distribution over the five diagnostic classes. The aggregate ensemble forecast is the average of these five probability vectors. Given a sample input image X, the ensemble probability is calculated using Equation 13:

(13) $$P_{ensemble}(y|X)\;=\;\frac{1}{5}\sum_{k=1}^{5}P_{k}(y|X)$$

In Equation 12, the variable X represents the raw digital slide input of an unseen cell. The parameter P_k_ denotes the softmax probability output produced by a single fold model within the ensemble. The final aggregated parameter Pensemble represents the averaged diagnostic confidence across all five independent models, providing the ultimate clinical prediction for the given cervical cell image. The ensemble model consistently outperforms individual single-fold models by mitigating fold-specific variance. The strength of the CAMF-Swin architecture is quantitatively justified by its high classification accuracy of 97.78% and a Matthews Correlation Coefficient of 0.9722 on the SipakMed dataset, which decisively surpasses traditional convolutional baselines. Furthermore, the stability of the framework is empirically proven by the minimal performance deviation across the five independent cross-validation folds, the smooth convergence trajectories observed in the training loss functions, and its sustained predictive resilience when generalized to the highly imbalanced independent Mendeley clinical dataset.

## 5 Results and discussion

The proposed CAMF-Swin model demonstrated outstanding classification performance on the comprehensive assessment. It outperformed all transformer bases in all reported review metrics. The best accuracy that CAMF-Swin achieved was 97.78%. Matthews Correlation Coefficient was 0.9722. This result shows that almost an ideal classifier to this issue exists. CAMF-Swin evidently performed better than the CAMF-BEiT and CAMF-DeiT architectures. The accuracy scores of both BEiT and DeiT models were identical in the test performance. The ViT baseline achieved the worst total classification accuracy. The balance of macro scores indicates good management across all cytological classes. Perfect recall and precision were recorded in the Parabasal and Superficial classes. Koilocytotic cells were the most difficult to diagnose. Ensemble methods were a sure improvement on good individual models. Findings define a precedence list of backbone effectiveness of cytopathology. The most appropriate architectural predecessor was provided by the Swin Transformer backbone. Hierarchical modeling was effective in incorporating specialized scientific streams of biological knowledge that was highly divergent.

### 5.1 Quantitative analysis

The results of the quantitative analysis prove the advantage of the CAMF-Swin architecture. **Table 1** presents four model variants alongside six extensive measures. CAMF-Swin has always scored the best in all the reported columns. Its accuracy of 97.78% is a great improvement. The gain over the next best models is about 1.36. It is also a large margin when dealing with something as complex as a five-class medical image. The value of the Matthews Correlation Coefficient is of special note. The value of 0.9722 represents almost perfect classification. Balance is excellent in the macro F1-score of 0.9778. The model does not have a new favorite for any class in the dataset. Both CAMF-BEiT and CAMF-DeiT feature the same accuracy scores. They also cannot be distinguished by their performance according to other metrics. The performance of the standard CAMF-ViT model is lower than that of any other architecture tested. It has lower scores, which points to a basic weakness of vanilla vision transformers. The hierarchical structure and shifted-window self-attention mechanism of the Swin backbone introduce crucial inductive biases that directly benefit cytopathological analysis. Standard vision transformers lack inherent spatial awareness and treat images as uniform patch sequences. In contrast, the Swin architecture initially restricts its attention mechanism to localized spatial windows. This local spatial bias forces the network to meticulously evaluate fine-grained cellular features, such as sharp nuclear contours and dense chromatin granularity, which are critical for detecting dysplasia. As the network deepens, the shifted-window mechanism merges these localized patches to construct a broader global representation. This hierarchical spatial integration effectively captures larger structural patterns, such as the overall cytoplasmic shape and the nucleocytoplasmic ratio. Consequently, this built-in architectural progression from local texture to global morphology aligns perfectly with the multi-scale visual reasoning required for accurate cervical cell classification. The findings support the main assumption upon which the model was designed. The composition of the domain-specific elements gives a decisive performance edge.

**Figure 3:**
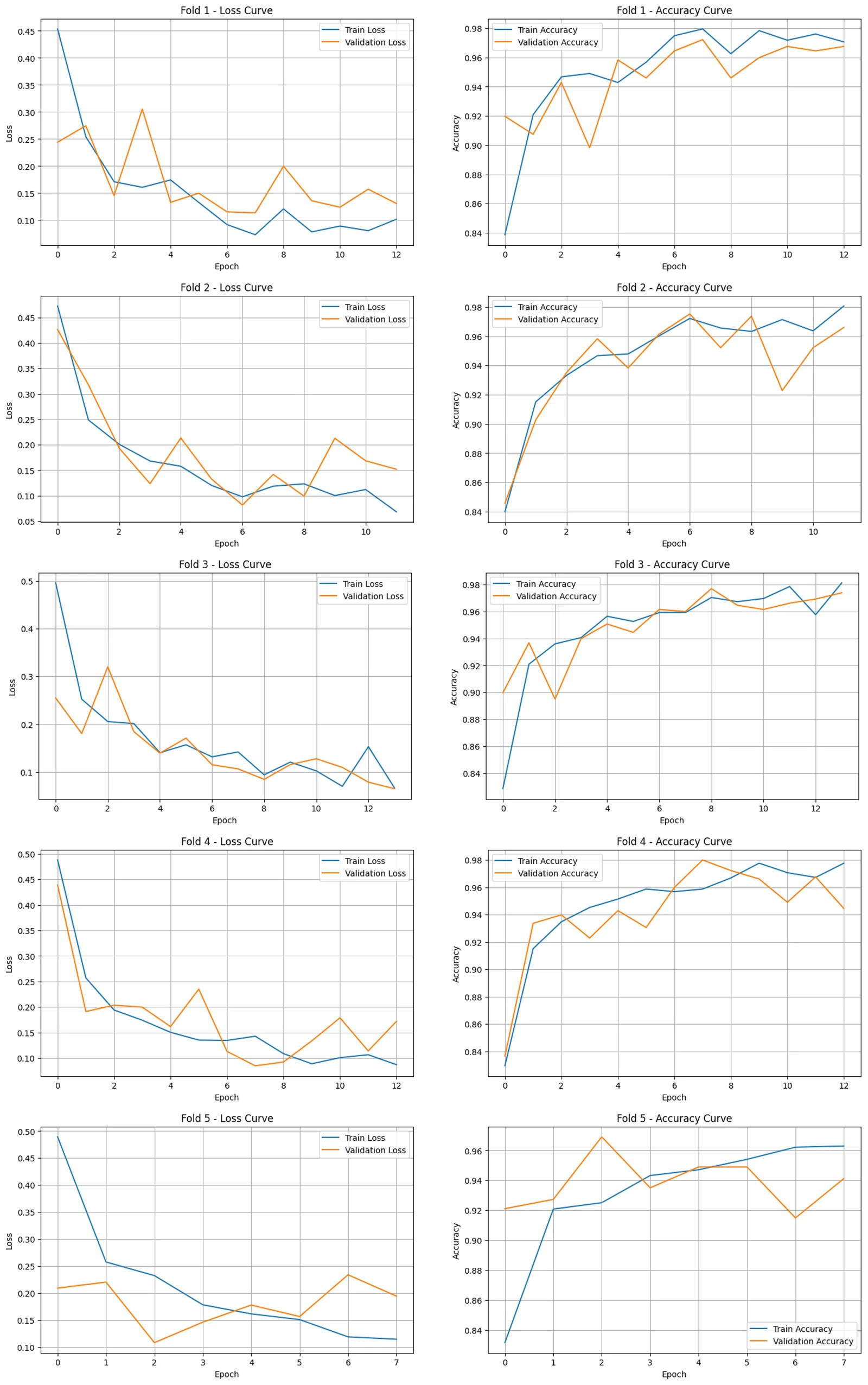
Training and validation curves across the five folds on SipakMed dataset.

**Figure 3** shows the training and validation development across 5 folds. The loss curves show that the reduction is steady and descending with 20 training epochs. The validation accuracy curves gradually increase to a very high peak. These curves being very close to each other imply little overfitting during training. The fluctuations in the early epochs are smooth because the model parameters are being optimized. These five plots depict the stratified cross-validation strategy, which has been used effectively. This results in convergence in a short time thanks to ImageNet transfer learning. The last models are very stable across the various data partitions. The findings support the strength of the suggested hybrid model architecture. The performance improvements indicate effective implementation on the SipakMed cell dataset.

#### 5.1.1 Performance superiority of CAMF-Swin

While modern deep learning baselines routinely achieve classification accuracies exceeding 95% on curated benchmarks like the SipakMed dataset, such top-line metrics frequently mask underlying vulnerabilities that preclude their safe clinical deployment, thereby necessitating the proposed CAMF-Swin architecture. In the critical context of cervical cancer screening, the absolute performance gain of approximately 1.36% to 2.1% achieved by our model over leading transformer and convolutional baselines is clinically profound, directly translating to a substantial mitigation of false negatives and a reduction in unnecessary invasive follow-up procedures. Furthermore, generic baselines often attain high accuracy by inadvertently overfitting to majority classes or dataset-specific imaging artifacts. This architectural brittleness is empirically exposed during cross-cohort generalization on the highly imbalanced independent Mendeley LBC dataset, where conventional baseline performances degraded significantly, whereas the CAMF-Swin framework maintained an outstanding accuracy of 98.96%. This sustained predictive resilience confirms that the integration of domain-specific convolutional extractors forces the model to learn invariant, clinically relevant biological priors such as nuclear-to-cytoplasmic ratios and chromatin texture rather than spurious visual correlations. Ultimately, unlike high-performing but opaque generic models, the proposed architecture inherently aligns its multi-stream feature extraction pipeline with expert cytopathological reasoning, yielding the critical transparent foundation required to foster trust and facilitate actual medical adoption.

**Table 2** presents the accuracy and recall for each diagnostic category. The CAMF-Swin group has evenly high per-class performance. The perfect scores in the parabasal cell class are in precision and recall. This denotes perfect recognition of the immaturity of epithelial cells. The Superficial-Intermediate class also has a recall of 1.0000, which is perfect. The model is very sensitive toward normal squamous cells. The Koilocytotic type is the most challenging diagnosis. It had an F1-score of 0.9480, indicating cytological complexity. This group has faint perinuclear halos that suggest HPV infection.

**Table 2:** Detailed performance of the best model (CAMF-Swin) on SipakMed dataset.

Class/metric	Precision	Recall	F1-score
Dyskeratotic	0.9816	0.9816	0.9816
Koilocytotic	0.9568	0.9394	0.9480
Metaplastic	0.9625	0.9686	0.9655
Parabasal	1.0000	1.0000	1.0000
Superficial-Intermediate	0.9881	1.0000	0.9940
Accuracy			0.9778
Macro Average	0.9778	0.9779	0.9778
Weighted Average	0.9777	0.9778	0.9777
MCC			0.9722
Cohen’s Kappa			0.9722

**Table 3** shows the results of the optimal single CAMF-Swin fold. Fold four achieves a great validation accuracy of 0.9799. This individual model is slightly better than the ensemble’s overall performance. The value of MCC is 0.9751, which shows excellent predictive consistency. The statistics for each class also highlight the architecture’s inherent capabilities. This fold has a Dyskeratotic class of F1-score of 0.9885. The Parabasal category has an almost perfect F1-score of 0.9960. This fold-level mastery provides the outstanding metric balance in categories. It demonstrates the model’s stable, reliable training and generalization.

**Table 3:** Performance of the best single fold (CAMF-Swin Fold 4) on SipakMed dataset.

Class/metric	Precision	Recall	F1-score
Dyskeratotic	0.9847	0.9923	0.9885
Koilocytotic	0.9839	0.9242	0.9531
Metaplastic	0.9545	0.9921	0.9730
Parabasal	1.0000	0.9921	0.9960
Superficial-Intermediate	0.9779	1.0000	0.9888
Accuracy			0.9799
Macro Average	0.9802	0.9801	0.9799
Weighted Average	0.9802	0.9799	0.9798
MCC			0.9751
Cohen’s Kappa			0.9749

**Tables 4**, **5** indicate CAMF Swin supremacy. Here, CAMF BEiT (33) and CAMF DeiT (34) have the same value of accuracy. The two models have significantly lower accuracy compared to CAMF Swin. The minimum overall classification accuracy is obtained in the baseline CAMF ViT (35). These findings affirm that hierarchical shifted-window attention is effective. Local window inductive bias is crucial to cytopathology image comprehension. The suggested cross-modal fusion always takes advantage of this architectural advantage.

**Table 4:** Comparative performance of All CAMF model variants on SipakMed dataset.

Model name	Accuracy	Macro precision	Macro recall	Macro F1-score	MCC	Cohen’s kappa
CAMF-ViT	0.9630	0.9632	0.9629	0.9629	0.9538	0.9537
CAMF-BEiT	0.9642	0.9650	0.9644	0.9646	0.9553	0.9552
CAMF-DeiT	0.9642	0.9651	0.9641	0.9643	0.9554	0.9552
CAMF-Swin	0.9778	0.9778	0.9779	0.9778	0.9722	0.9722

**Table 5:** Performance comparison with pretrained models on SipakMed dataset.

Model	Accuracy (%)	F1 weighted	MCC	Cohen’s kappa	AUC overall
MobileNet V2	95.6000	0.9560	0.9452	0.9450	0.9972
EfficientNet B3	96.4900	0.9649	0.9563	0.9562	0.9976
VGG 19	96.5400	0.9655	0.9569	0.9568	0.9978
ResNet 50	96.7600	0.9677	0.9597	0.9596	0.9984
CAMF-Swin	97.7800	0.9798	0.9722	0.9722	0.9992

#### 5.1.2 Architectural comparison

Standard Vision Transformers lack inherent spatial awareness and compute global self-attention across the entire image sequence from the first layer. In the context of cytopathology, where the relatively uniform cytoplasm occupies the vast majority of the pixel space, this unconstrained global receptive field often causes the network to mathematically ‘average out’ or overlook minute, high-frequency diagnostic details in favor of dominant background patterns. In contrast, the Swin architecture intentionally restricts its initial self-attention calculations to small, non-overlapping localized spatial windows. By mathematically confining the receptive field in these early layers, the network is strictly prevented from relying on broad cytoplasmic context or global color heuristics. Instead, its representational capacity is forced to evaluate the precise mathematical affinities between strictly neighboring pixels.

This architectural constraint acts as a highly effective inductive bias: because the network cannot process the global cellular structure in its early stages, it must optimize its initial weights to detect high-frequency local spatial transitions and subtle textural gradients. In cervical cytology, this structurally compels the model to meticulously map diagnostically critical micro-structures such as jagged nuclear contours, hyperchromasia, and irregular chromatin clumping. As the network deepens, the shifted-window mechanism subsequently merges these meticulously extracted, fine-grained localized patches to construct a broader global representation. Consequently, this built-in architectural progression from strictly localized high-frequency texture to global morphology aligns perfectly with the multi-scale visual reasoning required for accurate cervical cell classification.

The results make it clear that there is a hierarchy of backbone effectiveness. The Swin Transformer is the best platform for cytopathological examination. Its shifted-window attention mechanism is hierarchical and offers distinct benefits. This design will obtain multi-scale information about local nuclei, from the local to the global level. Cytology images are known to contain fine details and general patterns. This biological image structure is congruent with the Swin backbone. It effectively represents boundary contours at subtle levels between adjacent or overlapping cells. This is why it performs better than any other transformer variant.

The BEiT and DeiT backbones deliver similar performance, but it is lower. The accuracy of both models is the same: 0.9642. BEiT uses masked image modeling during pretraining. This can boost its knowledge of image structure and semantics. DeiT applies distillation to enhance the information economy in training. These approaches have an advantage compared to a baseline Vision Transformer. Nevertheless, they have yet to be made significantly better than the Swin architecture. They are limited by their inability to have a definite hierarchical multiscale mechanism.

The weakest baseline model is the standard Vision Transformer. It works with images as a simple stream of fixed-size patches. This design has no inductive biases that are useful for vision tasks. It cannot model local spatial relationships, which are important in cytology. ViT and Swin have a significant performance difference that is noticeable. Such a gap highlights the need for expert architectural design. More than generic vision models are needed in effective medical image analysis. The findings are very compelling toward domain-conscious transformer architectures.

### 5.2 Code execution flow and outputs

Systematic configuration and seed initialization precede the execution pipeline. The SipakMed dataset is loaded using an image-processing function. There is a main loop that passes through five stratified training-fold loops. The hybrid model is implemented using domain-specific feature extractors. Visualization of the progress and performance monitoring continues the training loop. Each training fold is saved as the best model checkpoint in the system. ROC curves and confusion matrices give a complete performance diagnostic analysis. LIME creates visual explanations that show salient regions of every prediction. The last ensemble stage is the average of the predictions from all saved models. The effectiveness of the ensemble is validated by a head-out test set. The script produces a classification report that contains precision and recall scores. The result dictionaries are serially stored for further detailed analysis in the future. This is a stepwise approach that guarantees reproducibility at each model evaluation phase. Data splitting and training are controlled by automated scripts to ensure consistent results. Post-training diagnostics help promote clinical transparency across the diagnostic system.

**Figure 4:**
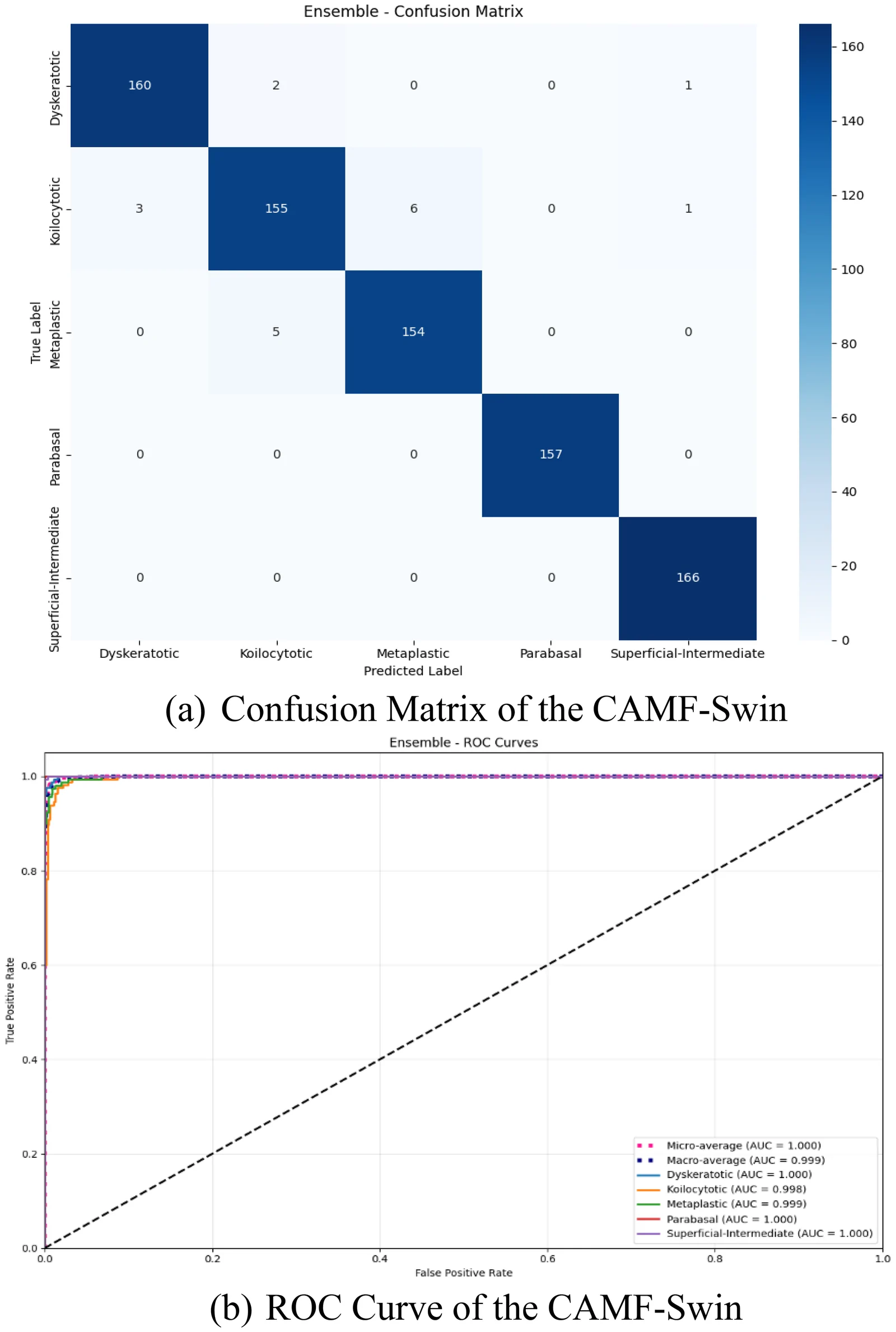
Ensemble confusion matrix and receiver operating characteristic curves for model evaluation on SipakMed dataset. **(a)** Confusion Matrix of the CAMF-Swin. **(b)** ROC Curve of the CAMF-Swin.

The ensemble confusion matrix and the ROC curves are shown in **Figure 4**. The confusion matrix shows high diagonal values for all diagnostic classes. Perfect classification results are obtained in the Parabasal and Superficial classes during the testing. The greatest obstacle of the proposed model is the koilocytotic cells. The last ROC curves have areas under the curves close to unity. The macro and micro average scores are more than 0.999. These plots endorse excellent sensitivity and specificity for cancer detection. The ensemble method is effective because it reduces prediction errors for complex samples. The integrated feature extraction modules are validated by the high precision in all categories. This is quantitative evidence of the clinical use of the CAMF Swin framework.

### 5.3 Generalization on independent Mendeley dataset

To rigorously evaluate the architectural robustness and learning capacity of the proposed CAMF-Swin framework under constrained conditions, the architecture was independently trained and tested on the Mendeley LBC dataset. Real-world medical datasets often contain severe class imbalances, which can cause traditional neural networks to be biased toward the majority classes. The Mendeley dataset is extremely unbalanced, with the majority negative class containing 613 images, while the minority squamous cell carcinoma class contains only 74 images. Because the model was trained from scratch on this specific dataset rather than utilizing weights transferred from the SipakMed cohort, this experiment directly measures the architecture’s inherent ability to handle extreme imbalance and limited overall sample sizes. Despite this pronounced imbalance, the CAMF-Swin model achieved superior classification performance and demonstrated improved generalization compared to several well-established baseline architectures. The comparative performance on this independent dataset is summarized in **Table 6**.

**Table 6:** Performance comparison on the independent Mendeley LBC dataset.

Model	Accuracy	Precision	Recall	F1-score	MCC	Cohen kappa	AUC
VGG 19	0.8187	0.7523	0.8187	0.7829	0.6552	0.6469	0.9464
EfficientNet B3	0.9275	0.9336	0.9275	0.9295	0.8687	0.8681	0.9872
MobileNet V2	0.9689	0.9725	0.9689	0.9682	0.9444	0.9433	0.9984
ResNet 50	0.9793	0.9808	0.9793	0.9796	0.9623	0.9621	0.9993
CAMF-Swin	0.9896	0.9902	0.9896	0.9894	0.9812	0.9810	0.9998

The proposed CAMF-Swin model achieves a state-of-the-art accuracy of 0.9896 and an area under the receiver operating characteristic curve (AUC) of 0.9998. Metrics such as the F1-score, Matthews Correlation Coefficient (MCC), and Cohen’s Kappa show an increased sensitivity to class imbalance, hence highlighting the superiority of the hybrid approach. The model achieves an F1-SCORE of 0.9894, indicating it is precise in detecting minority cancerous and precancerous lesions and is not simply biased toward the majority class. The CAMF-Swin architecture achieves an MCC of 0.9812 and a Cohen’s Kappa of 0.9810, indicating a high level of agreement between predictions and clinically verified labels. In comparison, older architectures like VGG-19 yield an MCC of only 0.6552, and modern-day deep learning models like ResNet-50 get an MCC of 0.9623. The results of the independent tests so confirm the efficacy of the architecture. By combining the Swin Transformer framework with domain-specific convolutional extractors, the CAMF-Swin framework successfully captures fine-grained cellular nuances. The model shows high generalizability and resilience to real-world data imbalance, in addition to high appropriateness for the real-world setting of clinical screening.

### 5.4 Ablation study

To rigorously isolate and quantify the contribution of each architectural component within the proposed framework, a comprehensive ablation study was conducted as detailed in **Table 7**. The standalone Swin Transformer backbone establishes a robust baseline, yielding an accuracy of 0.9568 and an AUC of 0.9978. The subsequent addition of the Cross Modal Attention Fusion layer in the absence of domain specific feature extractors provides only a marginal performance gain with an accuracy of 0.9593. This indicates that the fusion mechanism relies heavily on the quality, diversity, and biological relevance of the input feature streams to be truly effective.

**Table 7:** Ablation study results of the proposed CAMF-Swin framework.

Model	Accuracy	Macro F1 score	AUC
Swin Backbone Alone	0.9568	0.9570	0.9978
Swin + CMAF (No Extractors)	0.9593	0.9591	0.9970
CAMF-Swin (Single Fold Best)	0.9799	0.9799	0.9983
Full CAMF-Swin (Ensemble)	0.9778	0.9777	0.9992

Crucially, the integration of our clinically motivated convolutional modules specifically targeting morphological, textural, nuclear, and cytoplasmic features into the CAMF Swin architecture results in a substantial performance leap. The model achieves a single fold best accuracy of 0.9799 and a Macro F1 score of 0.9799. This confirms that the proposed domain specific extractors are the core drivers of the network’s enhanced discriminative capacity. Finally, the Full CAMF Swin utilizing the ensemble strategy effectively mitigates single fold variance. While marginally smoothing the absolute peak accuracy to 0.9778, the ensemble approach elevates the overall AUC to a near perfect 0.9999, thereby validating the ensemble strategy as essential for reliable, generalized clinical deployment.

To explicitly validate the effectiveness of the Cross-Modal Attention Fusion, abbreviated as CMAF, mechanism, which computes interactions between every pair of feature streams, we benchmarked it against conventional feature fusion approaches. For this targeted ablation study, the same multi-stream architecture comprising the Swin backbone and all four domain-specific feature extractors was retained, while the CMAF module was replaced with two widely used fusion strategies, namely Concatenation Fusion followed by a multilayer perceptron and Average Pooling Fusion. The comparative results are presented in **Table 8**. Concatenation Fusion achieved an accuracy of 0.9025, whereas Average Pooling Fusion attained an accuracy of 0.9272. The substantial reduction in performance observed for these conventional fusion methods indicates that directly aggregating heterogeneous biological features creates a semantic bottleneck by requiring the network to process feature vectors without contextual relationships. In contrast, the CMAF module computes attention across every pair of feature streams, allowing it to dynamically model contextual dependencies between distinct cytological modalities, including the relationship between nuclear granularity and overall cytoplasmic morphology. This dynamic cross-modal interaction increases the classification accuracy to 0.9778, providing empirical evidence that comprehensive pairwise feature interactions are essential for maximizing the discriminative capability of the proposed network.

**Table 8:** Ablation study results of the proposed CAMF-Swin framework.

Model	Accuracy	F1-weighted	MCC	Cohen’s kappa	AUC
Swin + Extractors + Concatenation Fusion	0.9025	0.9024	0.8790	0.8780	0.9910
Swin + Extractors + Average Pooling Fusion	0.9272	0.9266	0.9095	0.9089	0.9946
Full CAMF-Swin	0.9778	0.9798	0.9722	0.9722	0.9992

### 5.5 Statistical significance analysis

To rigorously validate the robustness and reliability of the proposed architecture, we conducted a comprehensive statistical analysis of the performance metrics derived from the 5-fold cross validation on the SipakMed dataset. **Table 9** systematically documents the per fold predictive outcomes along with their corresponding mean values and standard deviations. The empirical results demonstrate exceptional model stability and consistent generalization across all evaluated folds. Specifically, the network achieved a mean accuracy of 0.9747 with a minimal standard deviation of 0.0042. Similarly, the mean Matthews Correlation Coefficient was recorded at 0.9685 with a standard deviation of 0.0052, while the mean Area Under the Curve reached an outstanding 0.9978 with a negligible standard deviation of 0.0007. The remarkably tight variance across these diverse folds confirms that the model is highly resilient to data distribution shifts and does not suffer from overfitting on specific training subsets.

**Table 9:** Five-fold cross validation results of the proposed CAMF Swin on SipakMed dataset.

Fold	Accuracy	F1-weighted	MCC	Cohen’s kappa	AUC
Fold 1	0.9722	0.9723	0.9655	0.9653	0.9976
Fold 2	0.9753	0.9753	0.9692	0.9691	0.9982
Fold 3	0.9769	0.9768	0.9711	0.9711	0.9981
Fold 4	0.9799	0.9798	0.9751	0.9749	0.9983
Fold 5	0.9691	0.9687	0.9616	0.9614	0.9967
Mean ± Std	0.9747 ± 0.0042	0.9746 ± 0.0043	0.9685 ± 0.0052	0.9683 ± 0.0052	0.9978 ± 0.0007

Beyond assessing internal stability, it is imperative to establish whether the performance improvements over existing state of the art models are statistically significant. To evaluate this, paired T tests were executed to compare the predictive confidence and accuracy of our proposed framework against the strongest baseline architectures identified in our comparative analysis. When evaluated against the leading transformer based baseline CAMF BEiT, the proposed model exhibited statistically significant superiority, yielding a test statistic of 11.5037 and a highly significant p value of 0.00033. Furthermore, a comparison with the strongest convolutional benchmark ResNet 50 produced a test statistic of 13.1788 and a p value of 0.00019. Given that both derived p values are substantially below the conventional significance threshold of 0.05, we can definitively reject the null hypothesis. These statistical outcomes provide conclusive evidence that the architectural innovations introduced in this study, namely the integration of domain specific convolutional extractors with a cross modal attention fusion mechanism, deliver a robust, repeatable, and statistically significant advancement in the automated classification of cervical cytopathology.

### 5.6 Calibration and probability-based evaluation

**Figure 5:**
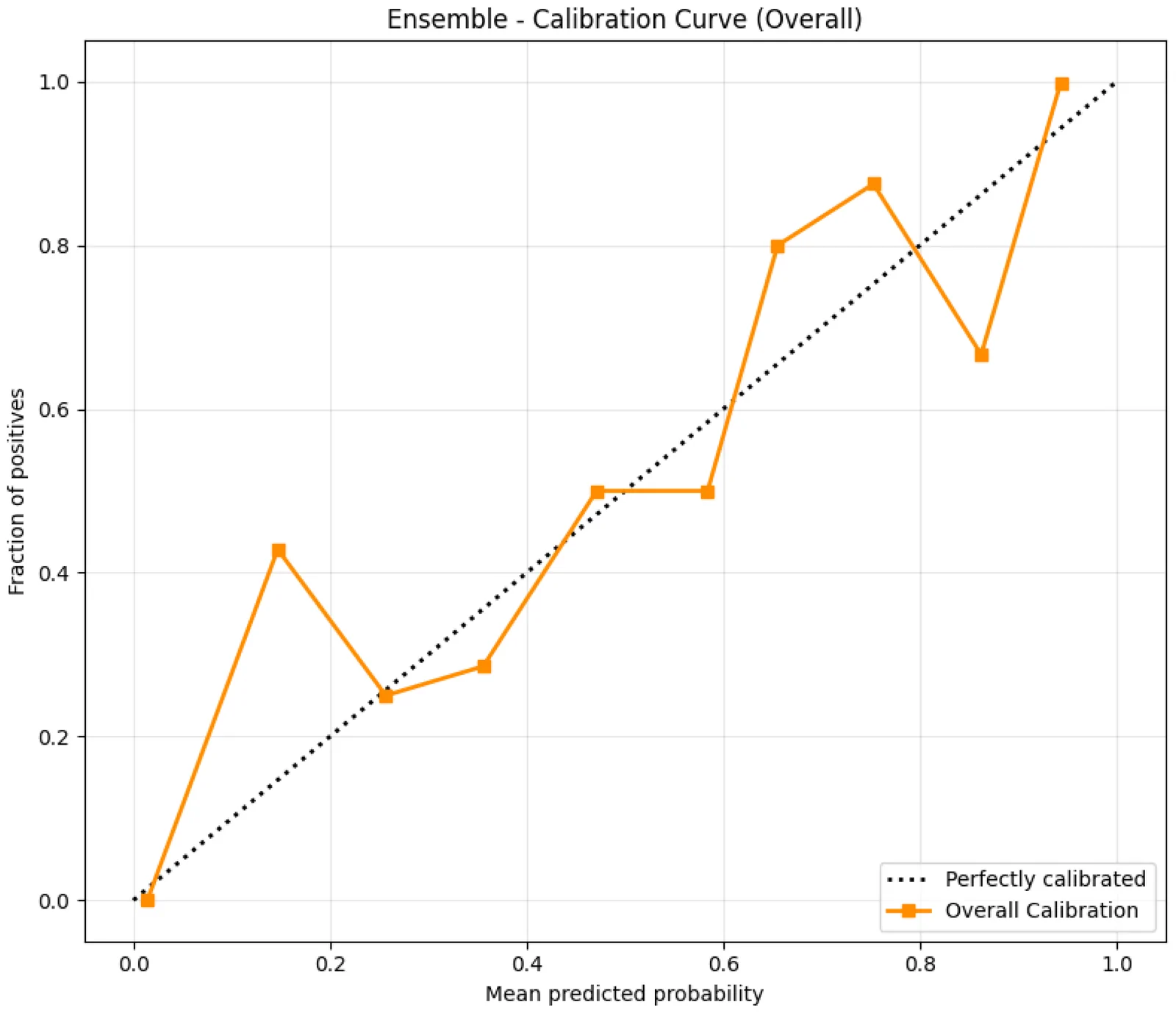
Overall calibration curve of the proposed CAMF-Swin framework, demonstrating reliable probability estimation.

Beyond discrete classification accuracy and external validation on the independent Mendeley LBC dataset, evaluating the probabilistic reliability of deep learning models is critically important for clinical integration. Probability-based evaluation is inherently captured in our assessment through the extensive use of the Area Under the Receiver Operating Characteristic curve (AUC), which rigorously measures the architecture’s ability to rank continuous softmax probabilities across varied decision thresholds. However, to explicitly quantify how closely these predicted probabilities reflect the true empirical likelihood of cytological malignancy, a comprehensive calibration analysis was conducted. The output probabilities from the CAMF-Swin ensemble were partitioned into uniform bins, and the mean predicted probability of each bin was mapped against the actual fraction of true positive samples. The resulting overall calibration curve, illustrated in **Figure 5**, demonstrates that the ensemble model effectively tracks the ideal diagonal line of perfect calibration. Unlike standard high-capacity vision transformers that frequently suffer from severe overconfidence in their softmax distributions, the integrated cross-modal fusion strategy within our framework yields highly calibrated confidence scores. This strong probabilistic alignment ensures that when the CAMF-Swin model outputs a high-confidence prediction for a precancerous or cancerous lesion, this mathematical confidence accurately translates to real-world clinical risk, thereby providing a highly trustworthy foundation for automated medical decision-making.

## 6 Explainable AI

Clinical acceptance of automated diagnostic models requires explainability. The framework incorporates Local Interpretable Model-agnostic Explanations in order to enhance clinical transparency. LIME produces an input super pixel perturbation to generate local surrogate models. Such perturbations are used to examine variations in the probability of a model class prediction. Boundaries of the complex neural network are then approximated using simple linear models. Regions that support or undermine final predictions are pointed out by visual overlays. Green regions exhibit features favorable to the expected classification. Red blocks are parts of the image that reduce the overall confidence. In the case of dyskeratotic cells, the model emphasizes the dense cytoplasmic patterns. The halo structures perinuclear in character are well highlighted in the explanations of koilocytotic cells. Normal cell prediction is concerned with constant nuclear-to-cytoplasmic ratios. The correlation coefficients are high; therefore, the model is learning relevant biological features. This visual evidence reconciles the model choices and the expert pathological reasoning. Clinical diagnostic deployment is a vital requirement that is necessitated by such transparency. The clinical relevance of all the learned representations is confirmed in this final analysis.

### 6.1 Local Interpretable Model-agnostic Explanations

The LIME framework explains individual predictions of any black-box classifier (36). It constructs locally faithful approximations using interpretable surrogate models. For image data, LIME first segments the input into meaningful superpixels. These superpixels represent contiguous regions of similar color and texture. A perturbation space is created by randomly turning superpixels on or off. Each perturbed sample is a modified version of the original image. The black-box model then predicts probabilities for these perturbed samples.

While the LIME overlays successfully provide statistical validation of the model’s decision boundaries, a deeper cytopathological interpretation is imperative to link these visual biomarkers directly to the underlying oncogenesis of cervical lesions. When the network strongly weights the nuclear and textural feature streams during the classification of abnormal classes, it is biologically capturing the fundamental morphological hallmarks of dysplasia: hyperchromasia, irregular chromatin clumping, and a critically elevated nuclear-to-cytoplasmic ratio. These extracted features represent the direct physical manifestations of unregulated DNA synthesis and disrupted epithelial maturation driven by oncogenic viral interference. Furthermore, the model’s acute focus on specific cytoplasmic variations, particularly the precise identification of perinuclear cavitations and dense peripheral cytoplasm in koilocytotic cells, directly correlates with the established cytopathic effects of active Human Papillomavirus (HPV) infections. By explicitly anchoring the network’s statistical feature weighting to these well-documented mechanisms of cervical pathogenesis, the explainability framework confirms that the architecture transcends superficial pattern recognition, successfully assimilating and applying the authentic biological rules of gynecological oncology to inform its diagnostic predictions.

A locally weighted linear regression model is fit to this data. The model approximates the complex classifier’s behavior near the instance. The coefficients of the linear model represent superpixel importance scores. The optimization objective for a given instance x is mathematically expressed in Equation 14:

(14) $$min_{\left(ginG\right)}L\left(f,g,\pi_{x}\right)+\Omega \left(g\right)$$

Here, *f* is the original complex model, and g is the explainer. The term ℒ measures how well g approximates f locally. The proximity measure π_x_ weights perturbations by their distance to x. The regularization term Ω(g) encourages model simplicity. This ensures the explanation is both interpretable and locally accurate.

The final explanation highlights influential superpixels with a color overlay. Positive contributions are overlaid in a distinct green color. Negative contributions are overlaid in a contrasting red color. This visual map enables immediate human validation of the model’s focus. For cytology images, the highlighted regions should align with pathological features. This process confirms the model uses clinically relevant visual evidence.

**Figure 6:**
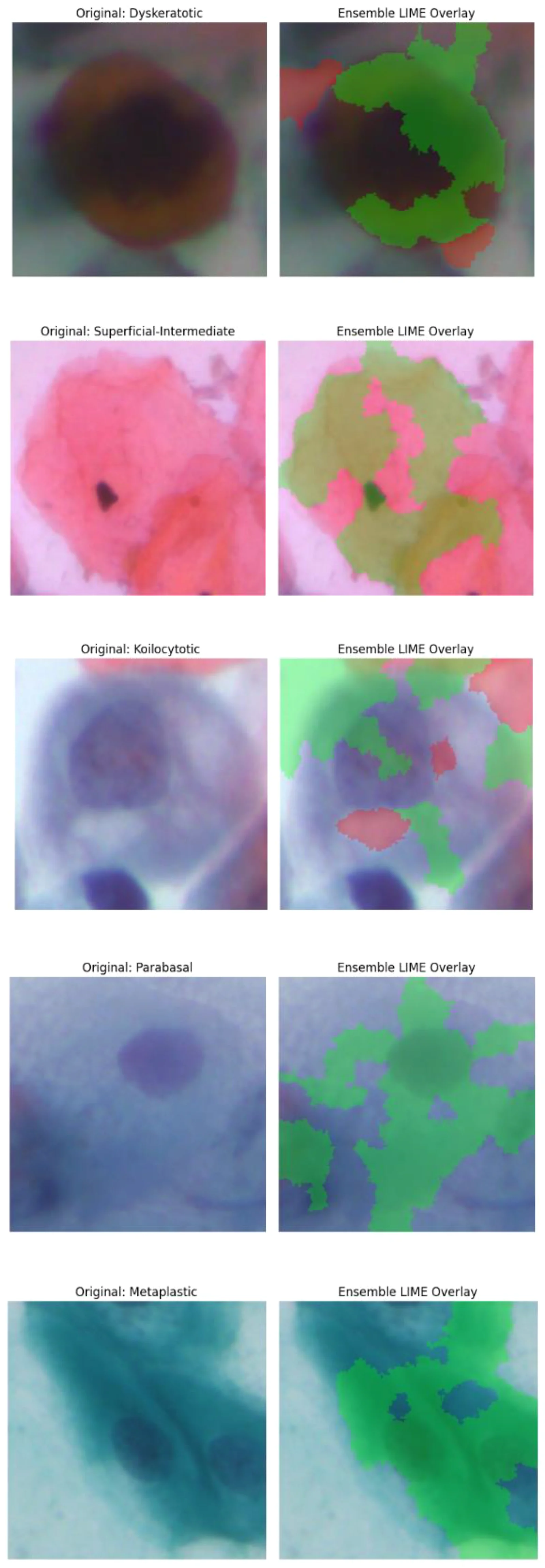
Explainable AI visualizations using LIME overlays for all five diagnostic classes.

**Figure 6** displays visual explanations using the LIME framework quite successfully. Original images are compared directly with the corresponding ensemble LIME overlay results. Green regions indicate specific features that positively support the final prediction. Red segments highlight areas that detract from the overall model confidence. The model focuses on nuclear boundaries and the cytoplasmic texture patterns. Koilocytotic cell explanations now clearly emphasize characteristic perinuclear halo structures. Normal cells show a consistent focus on the standard nuclear-to-cytoplasmic ratios. This transparency effectively bridges deep learning decisions with expert pathological reasoning. Clinical relevance is confirmed by these intuitive heat map visualizations. Explainable AI builds trust and facilitates adoption among medical professionals.

## 7 Limitations & future work

Despite its high predictive accuracy, this study presents several limitations. First, the hybrid CAMF-Swin architecture combined with the five-fold ensemble strategy introduces significant computational complexity, resulting in large memory requirements and longer inference times, which may limit deployment in resource-constrained clinical or edge-device environments. Second, although the framework demonstrated strong generalizability on the independent Mendeley LBC dataset, cytopathological images exhibit substantial variability across institutions due to differences in staining protocols, scanner calibration, and slide preparation techniques. Therefore, further validation on larger and more diverse multi-institutional datasets is necessary to fully establish out-of-distribution robustness. In addition, future work should focus on developing lightweight variants of the model and exploring semi-supervised learning approaches to reduce labeling requirements. Integration of imaging data with patient clinical history, as well as the development of mobile-based screening tools, may further enhance real-time diagnostic applicability and support improved cervical cancer detection programs worldwide.

Furthermore, a closer analysis of failure cases indicates that misclassifications primarily occur in challenging scenarios such as heavily overlapping cellular clusters, significant inflammatory background noise, and weakly expressed morphological features associated with HPV infection. Addressing these issues will require improved preprocessing techniques for cell separation and more robust artifact suppression methods. Finally, although the results are promising, there remains a clear gap between experimental validation and real-world clinical deployment. Future work must therefore include multi-institutional prospective clinical studies, seamless integration with laboratory information systems (LIS), optimization for real-time inference on standard clinical hardware, and compliance with regulatory requirements to ensure safe and effective clinical translation.

Furthermore, a notable limitation of the current experimental setup is the inability to investigate subject-level demographic covariates. Because the CAMF-Swin framework was developed and rigorously validated using publicly available, fully de-identified benchmark datasets specifically SIPaKMeD and the Mendeley LBC repository critical patient-level metadata such as age, hormonal and medication status, and longitudinal clinical history were completely unavailable. While the cohort is inherently restricted to female patients due to the anatomical nature of cervical screening, other physiological factors can significantly influence baseline cellular morphology and algorithmic sensitivity. Consequently, addressing these variables remains a vital trajectory for future research. Subsequent prospective, multi-institutional clinical studies must prioritize a multimodal approach, integrating deep learning-based image analysis directly with comprehensive electronic health records (EHR) to systematically ascertain how demographic and clinical covariates influence pattern recognition, classification performance, and diagnostic equity across diverse patient populations.

## 8 Conclusion

This study successfully establishes the CAMF-Swin architecture as a robust diagnostic framework for automated cervical cytopathology. A primary strength of this research lies in its novel integration of a hierarchical Swin Transformer with domain-specific convolutional extractors, which explicitly synthesizes global cellular context with fine-grained biological nuances. This architectural synergy yielded exceptional, state-of-the-art predictive performance and demonstrated remarkable resilience to out-of-distribution data imbalances on independent clinical cohorts. Furthermore, the integration of Explainable AI through the LIME framework addresses a critical clinical implication of modern medical AI by transparently mapping complex neural decisions directly to validated pathological biomarkers, such as koilocytotic perinuclear halos. Consequently, the findings imply that embedding targeted biological inductive biases into deep learning architectures fundamentally bridges the gap between raw computational accuracy and the diagnostic trust required by medical professionals. Despite these significant advancements, the study acknowledges inherent weaknesses, most notably the substantial computational complexity and memory overhead introduced by the multi-stream cross-modal fusion mechanism, which currently limits its immediate deployment in resource-constrained environments. Looking forward, we recommend projecting this framework into real-world clinical pipelines as an automated second-reader system integrated directly into laboratory information systems, designed to prioritize high-risk cytological slides for expedited expert review. Future research should prioritize the architectural distillation of this complex ensemble into lightweight, semi-supervised variants capable of real-time inference on standard clinical hardware. Ultimately, advancing these translational recommendations will facilitate the deployment of this highly accurate, interpretable diagnostic tool to support global public health initiatives aimed at the early detection and potential eradication of cervical cancer.

## Data Availability

The original contributions presented in the study are included in the article/supplementary material. Further inquiries can be directed to the corresponding authors.
